# Vector-borne transmission of *Besnoitia besnoiti* by blood-sucking and secretophagous flies: epidemiological and clinicopathological implications

**DOI:** 10.1186/s13071-015-1058-0

**Published:** 2015-09-04

**Authors:** Sándor Hornok, András Fedák, Ferenc Baska, Walter Basso, László Dencső, Gergely Tóth, Levente Szeredi, Tamás Abonyi, Béla Dénes

**Affiliations:** Department of Parasitology and Zoology, Faculty of Veterinary Science, Szent István University, Budapest, Hungary; Veterinary Authority, Miskolc, Hungary; Department of Pathology, Faculty of Veterinary Science, Szent István University, Budapest, Hungary; Institute of Parasitology, Vetsuisse Faculty, University of Zürich, Zürich, Switzerland; Veterinary Diagnostic Directorate, National Food Chain Safety Office (NFCSO), Budapest, Hungary

**Keywords:** *Besnoitia besnoiti*, Venereal, Transplacental, Vector-borne, Fly, Tick, Eradication

## Abstract

**Background:**

Bovine besnoitiosis has been recently diagnosed in a three-parted herd of 796 Aubrac and Charolais beef cattle in Hungary. A large scale serological, histological and molecular survey was initiated in order to uncover important factors in the local epidemiology of the disease.

**Findings:**

Blood samples were collected (three times from the whole herd, and repeatedly from selected animals) for serological screening by ELISA. In addition, various organs from aborted fetuses and newborn calves, skin and colostrum samples of seropositive heifers/cows, and ticks collected from the cattle were histologically and/or molecularly analysed for the presence of *Besnoitia besnoiti*.

All fetal and calf tissues, as well as colostrum and tick samples from cows were PCR negative. Based on ELISA results, only very few local cows seroconverted after mating with imported, infected bulls, and not necessarily as a consequence of this event. Among calves that were born to seropositive, imported cows and stayed with their mother until weaning at seven months of age, seroprevalence decreased significantly, but remained high. At the same time, 28 calves born from seropositive cows, but separated from their dams immediately after receiving colostrum, were successfully reared and remained uninfected.

Following a second herd-level screening, all Aubrac cattle (except for heifer calves) and all seropositive Charolais cows and bulls were culled. Manifestation of the disease is currently sporadic. Among chronically affected heifers two types of skin lesions were noted, and histological evaluation indicated marked distension of sweat gland ducts with membrane-bound structures (resembling cystozoites) in their contents.

**Conclusions:**

Transmission through natural mating, as well as transplacental, colostral and tick-borne transmission of *B. besnoiti* was either unlikely or did not occur. However, the risk for spreading of the infection was high, when calves stayed with their mother during suckling, and if animals were kept in the same stable (although physically separated) during the main fly season. Herd replacement and generation exchange (i.e. early weaning and artificial feeding) appear to be the successful strategies for the local eradication of bovine besnoitiosis. Adding to the already known mechanical transmission of *B. besnoiti* by blood-sucking flies, results of the present study suggest that secretophagous flies should also be evaluated as potential vectors of this coccidium species.

## Findings

### Background

*Besnoitia besnoiti* (Apicomplexa: Sarcocystidae) is a cyst-forming coccidian parasite that may cause severe lesions (with usually high morbidity, but low mortality) in cattle as intermediate hosts [1]. Wild bovids and probably other wild ruminants might also be susceptible to *B. besnoiti* infection [2]. As a unique example among cystogenic coccidia, the main transmission route of *B. besnoiti* appears to be vector-borne, i.e. mechanically by blood-sucking dipterans (tabanid horse flies, stable flies), although it is also possible iatrogenically (with intra- or hypodermic needles) and most likely through close contact between animals [3]. Bovine besnoitiosis has been endemic in South-Western Europe for more than a century, but a significant expansion of its geographical distribution, to other parts of the formerly endemic countries and countries neighboring France, was observed only during the last decade [1]. More recently, the presence of *B. besnoiti* was recognized in Hungary (for the first time in Central-Eastern Europe: [4]), Croatia [5] and Greece [6]. In the latter countries the epidemiological situation may become similar to that in Italy, where bovine besnoitiosis is not regarded as endemic, but rather persists in independent foci of infection [7].

This eastward spreading of a vector-borne disease is unusual in the era of global warming and climate change, when scientists expect northward expansion of the habitats of vectors, and consequently of the occurrence of associated pathogens [8]. However, in the case of bovine besnoitiosis competent vectors are indigenous in most parts of Europe, and therefore for the emergence of this disease in Central-Eastern Europe only cattle trading can be blamed. Accordingly, the establishment of bovine besnoitiosis in a three-parted beef cattle herd (in one location) in Hungary resulted from the import of Aubrac heifers and bulls from France in 2011–2012 [4]. The aim of the present study was to investigate the epidemiology and clinico-pathological manifestation of the disease in the relevant location, including attempts of eradication by herd replacement, generation exchange and selection with culling. The study was initiated in the hope that the results will help to plan and install proper control measures for preventing further spread of the infection not only in Hungary, but in a broader geographical range of Europe.

### Methods

#### Herd management

**Fig. 1:**
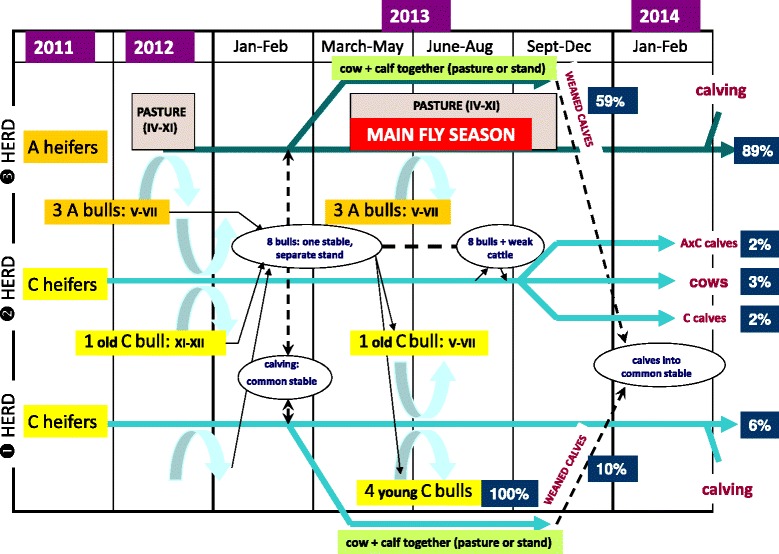
Herd management and temporal significance of ecological factors relevant to bovine besnoitiosis. Percentages next to a category show the proportion ELISA positive animals at the first herd level screening (November, 2013). Curved arrows indicate mating of cows with the bull shown at the basis of the arrow. Abbreviations: A - Aubrac breed, C - Charolais breed, Roman numerals – months

The study herd consisted of three parts: (1) and (2) including original, locally kept Charolais cattle, and in (3) Aubrac cows and bulls imported in 2011–2012 (Fig. 1). These three groups were grazing separate pastures (approx. 1–2 km apart). Natural mating was scheduled in two periods (November-December and May-July), out of which the bulls were stabled, close to weak or diseased cows separated from the others (Fig. 1). Aubrac and Charolais cows were stabled together for calving during the winter. Between April and November, the cows and their calves were turned out to the pastures in the morning and afternoon each day, while spending the hottest hours (around noon) in a common stand. Calves, following weaning around seven months of age, were stabled collectively (Fig. 1).

#### Sample collection

Non-anticoagulated blood samples were collected from all cattle in the herd for serological screening three times: (1) first in November, 2013 for the evaluation of the epidemiological situation and the potential significance of transmission routes; (2) then in March-April, 2014 and (3) in September, 2014 for the selection of seropositive animals for culling (Table 1). Additional blood samples were collected for serological analysis from cows that showed clinical signs (leg oedema, then skin lesions) characteristic of bovine besnoitiosis.

**Table 1:** Samplings in chronological order, showing the number of seropositive cattle per all tested

		2013	2014			2015
Breed	Target group	November	February	March-April	September	February
Charolais	Herd #1 and #2	31/559	-	26/589^3^	30/564	-
Aubrac	Herd #3: bulls, cows	191/237	-	188/237	-	-
	Herd #3: heifer calves	-	0/15^1^, 13/14^2^	-	4/29	0/29

^1^on the day of birth

^2^at one week of age

^3^discounting newborn calves

In February 2014, 29 calves born from seropositive Aubrac cows were separated from their mother either before (one calf) or immediately after ingestion of colostrum (28 calves), followed by artificial feeding. These calves were blood-sampled for serological analysis at or shortly after birth (15 and 14 of them, respectively), then at seven months and at one year of age (February and September, 2014, February 2015: Table 1).

Tissue samples (blood, distal tendons of the leg, nasal conchae, lungs, skin, trachea, liver, kidney) were collected for histology and PCR from three aborted fetuses and two newborn calves delivered by seropositive Aubrac cows in January and February, 2014. Biopsy was done on two Charolais cattle (herd No. 1: cows B1, B2) during acute besnoitiosis (from the swollen, distal part of the leg), then during chronic besnoitiosis (from skin lesion of the neck), in May and August 2014, respectively. Both animals were seropositive. Additionally, colostrum samples were taken from seven seropositive Aubrac cows in February, 2014. Ticks (*Ixodes ricinus*: 11 males, 12 females; *Dermacentor reticulatus*: 12 males, 11 females) were collected from seropositive and seronegative cows in September, 2014 (one male/female tick per host). These were mechanically cleaned of any tissue remnants, and then stored in 70 % ethanol until evaluation.

#### Sample analyses

All serum samples were screened with a commercially available ELISA (PrioCheck Besnoitia Ab 2.0, Prionics, Zurich, Switzerland). The ELISA used in the present study was shown to have a 98.8 % specificity [9]. From the optical density (OD) values of the controls the percentage positivity (PP%) value was calculated according to the manufacturer’s instructions. Samples with PP values above 23 % (cut-off) were regarded as positive.

Biopsy and histology was performed as described [4, 10].

DNA was extracted from fetal/calf tissue samples, colostrum (200 μl) and individual ticks with QIAamp DNA Mini Kit (QIAGEN, Hilden, Germany). With these samples a real-time PCR, based on sequence amplification of the internal transcribed spacer region 1 (ITS-1) of the rRNA gene of *B. besnoiti*, was performed [11].

#### Statistical analyses

Exact confidence intervals (CI) for the prevalence rates were calculated at the level of 95 %. Seroprevalence data at the first sampling (November, 2013) were compared using Fisher’s exact test, and differences were regarded significant when *P* ≤ 0.05.

### Results and discussion

#### The potential role of venereal, transplacental and colostral transmission

Only 5 of 159 of Charolais cows (3 %, CI: 1–7.2 %) that mated with infected Aubrac bulls were found seropositive (herd No. 2: Fig. 1). Considering that even this low seropositivity may have resulted from temporary stabling of a few weak cows close to Aubrac bulls (Fig. 1), transmission through natural mating (via mucosal contact of the genitalia) in the present study appeared to be absent or to have very low significance. This is in contrast to some other observations emphasizing the potential (but never verified) importance of mating in the spreading of bovine besnoitiosis [1].

Histological and molecular evaluation of all organs/tissues of three aborted fetuses and two newborn calves (delivered by seropositive cows) yielded negative results for *B. besnoiti*. Fifteen calves born from seropositive cows were seronegative immediately after birth (based on pre-colostral blood samples). In additon, there was a significant (*P* < 0.0001) difference between the seroprevalence in the case of Aubrac cows compared to their calves at nine months of age (at the first sampling in November, 2013), i.e. 149 of 168 cows (89 %, CI: 82.9–93.1 %) and 39 of 66 calves (59 %, CI: 46.3–71.1 %) were seropositive (Fig. 1). Maternal antibodies most likely did not interfere with the latter results, because these are eliminated by 6–8 months of age [12]. According to the above, the transplacental or periparturient transmission of *B. besnoiti* (e.g. if mucosal cysts in the female genitalia rupture during birth) is either unlikely or does not occur. These findings are consistent with those reported for *B. caprae* [13].

In addition, all seven colostral samples of seropositive Aubrac cows were PCR negative. Thus, shedding of *B. besnoiti* with the colostrum was not detected.

#### The potential role of mechanical transmission

According to the above, the relatively high (59 %) seropositivity among weaned Aubrac calves may not be attributable to vertical transmission, but rather to horizontal transmission by mechanical vectors (blood-sucking flies), because calves were kept close to their mother cows (especially during suckling) during the main fly season (Fig. 1). This may have resulted in the significant (*P* < 0.0001) difference between the seropositivity among Aubrac (39 of 66: 59 %, CI: 46.3–71.1 %) vs. Charolais calves (6 of 63: 10 %, CI: 3.6–19.6 %), corresponding to the serological status of mother cows in both relevant herds (No. 1, 3: Fig. 1).

The reported significance of mechanical (fly-borne) transmission [1] is supported by further data in the present study, i.e. all four young Charolais bulls seroconverted after being stabled close to the infected Aubrac bulls at the beginning of the main fly season in April, 2013 (herd No. 1: Fig. 1). On the other hand, Aubrac and Charolais cows were stabled together for calving during the winter at the beginning of 2013, i.e. out of the fly season, but the seroprevalence among Charolais cows remained low (herd No. 1: 12 of 187 seropositive, i.e. 6 %, CI: 3.4–10.9 %). This could be explained by the absence or low significance of other means of mechanical transmission (e.g. by mucosal or skin contact, or sharp objects as postulated by some authors, e.g. in [1, 13]).

On account of the potential presence of hard ticks (Acari: Ixodidae) on stabled cattle in the wintertime, it was suggested that males (while transferring between animals during intermittent blood-sucking) may also be mechanical vectors of *B. besnoiti* [14]. To test this hypothesis, in the present study ticks removed from seropositive and seronegative cattle were molecularly analysed for the presence of *B. besnoiti* DNA. However, all were PCR negative. Therefore the postulated tick-borne transmission of *B. besnoiti* could not be confirmed.

#### Clinico-pathology of acute and chronic besnoitiosis in the herd

**Fig. 2:**
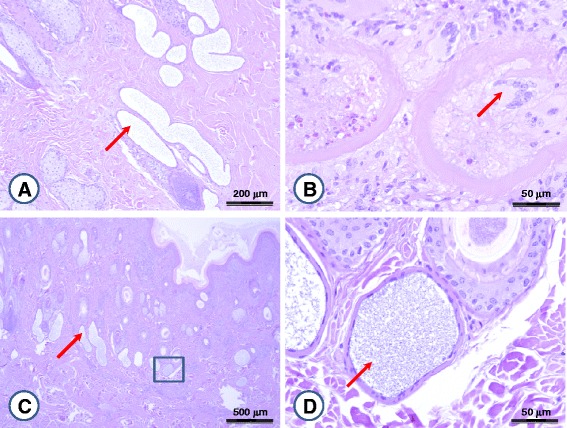
Lesions associated with acute (**a**) and chronic (**b**-**d**) bovine besnoitiosis. Arrows indicate (**a**, **c**) distended sweat gland ducts, (**b**) giant cell in a former cyst, and (**d**) membrane-bound structures, resembling cystozoites, in sweat gland tubules. The insert in (**c**) is enlarged on (**d**)

In tissue sections from two acute cases, developmental stages (tachyzoites) were not seen in endothelial cells. The main findings were lymphoid hyperplasia (with plasmocyte infiltration), generalized skin oedema and dilation of sweat gland tubules (Fig. 2a). Except for the latter, these are consistent with those already reported [15].

For chronic stage besnoitiosis, two types of lesions could be distinguished. In the case of typical clinical manifestation (Charolais cow “B1”: skin thickening, wrinkling and alopecia: so-called elephant skin disease) numerous large cysts were seen in skin sections. Some of the cysts showed signs of resolving, i.e. cystozoites were cleared up and replaced by histiocytes, eosinophilic granulocytes and giant cells (Fig. 2b).

Cow “B2” and three further Charolais cows presented with a different type of lesion, macroscopically characterised by thin (semi-transparent) skin and distension of superficial blood vessels. In corresponding tissue sections only few and smaller, shrunken tissue cysts were seen, surrounded by markedly dilated ducts of sweat glands (Fig. 2c). In the contents of sweat gland tubules membrane-bound, crescent shape structures were seen, resembling (and in the size range) of cystozoites (Fig. 2d). Unfortunately, due to the method of fixation, the fine structure of these could not be discerned. However, this finding raises the possibility that cystozoites may reach the exterior not only by rupturing of mucosal cysts [1, 16], but also through sweating. This may theoretically provide the means of transmission by secretophagous flies (worthy of future investigation), if cystozoites are delivered onto the conjunctiva or mucosal surfaces which they are able to penetrate [13]. Tachyzoites of *B. besnoiti* were shown to be present in lachrymal secretions, based on which their potential spread by secretophagous flies was also postulated [17].

#### Attempts of eradication

Concerning the methods of eradication (to regain the status “free of bovine besnoitiosis”), (1) herd replacement (emptying the farm) was possible only in case of herd No. 3 (Fig. 1). After calving and the second herd screening (March-April, 2014) the majority of adult Aubrac cattle were found to be still seropositive (Table 1), therefore all animals in this herd (except heifer calves) were culled.

In the case of newborn Aubrac heifer calves, a second method of eradication, a small scale (2) generation exchange was attempted. This implied separation of calves from their seropositive mother cow either prior to or after receiving colostrum. One calf was reared with pre- and 28 calves with post-colostral weaning. Concerning the latter group, at birth 15 of the calves could be tested, and all were seronegative. Other (i.e. 13) calves in this group were serologically evaluated at approx. one week of age, and all were seropositive. At seven months of age four of the 28 calves were still seropositive, but by one year of age all became seronegative (Table 1). These findings are consistent with acquiring passive immunity from mother cows, followed by gradual elimination of maternal antibodies by 6–8 months of age as reported [12]. The calf with pre-colostral weaning remained seronegative in all above tests. Therefore this method of eradication was deemed successful.

In addition, (3) selection with culling of seropositive animals was also attempted. All Charolais cattle that were seropositive at the first or second herd screening (Fig. 1, Table 1) were culled prior to the main fly season, i.e. in March, 2014. However, six months later (September, 2014: Table 1) there were still 30 out of 564 seropositive cows in the herd (5 %, CI: 3.6–7.5 %). Although this level of seroprevalence is much lower than reported for regions with endemic stability (after three years, as in the present case, it may be in excess of 80 %: [3]), this result together with the sporadic occurrence of clinical cases (up to seven per year) justifies further monitoring. Failure of complete eradication with selection can be explained by the presence of low number of infected, but (at the time of blood sampling) seronegative animals.

### Conclusions

Venereal, transplacental, colostral and tick-borne transmission of *B. besnoiti* was either unlikely or did not occur. However, the risk for spreading of the infection was high, when calves stayed with their mother during suckling, and if animals were kept in the same stable (although physically separated) during the main fly season. Herd replacement and generation exchange (i.e. early weaning and artificial feeding) appear to be the successful strategies for the local eradication of bovine besnoitiosis. Adding to the already known mechanical transmission of *B. besnoiti* by blood-sucking flies, results of the present study suggest that secretophagous flies should also be evaluated as potential vectors of this coccidium.
